# Fluidic Self-Assembly on Electroplated Multilayer Solder Bumps with Tailored Transformation Imprinted Melting Points

**DOI:** 10.1038/s41598-019-47690-8

**Published:** 2019-08-05

**Authors:** Mahsa Kaltwasser, Udo Schmidt, Lars Lösing, Shantonu Biswas, Thomas Stauden, Andreas Bund, Heiko O. Jacobs

**Affiliations:** 1 Fachgebiet Nanotechnologie, Technische Universität Ilmenau, Gustav-Kirchhoff-Strasse 1, Ilmenau, D-98693 Germany; 2 Fachgebiet Elektrochemie und Galvanotechnik, Technische Universität Ilmenau, Gustav-Kirchhoff-Strasse 6, Ilmenau, D-98693 Germany; 3 California NanoSystems Institute, University of California, Santa Barbara, CA 93106 USA

**Keywords:** Metals and alloys, Electronic devices

## Abstract

This communication presents fluidic self-assembly of Si-chip on a sequentially electroplated multilayer solder bump with tailored transformation imprinted melting points. The multilayer solder bump is a lead free ternary solder system, which provides a route to transform the melting point of interconnects for applications in solder directed fluidic self-assembly. The outermost metal layers form a low melting point Bi_33.7_In_66.3_ solder shell (72 °C). This solder shell enables fluidic self-assembly and self-alignment of freely in water suspended Si-dies at relatively low temperature (75 °C) leading to well-ordered chip arrays. The reduction of the free surface energy of the shell-water interface provides the driving force for the self-assembly. The lowermost metal layer is a high melting point solder and acts as a core. After the self-assembly is complete, a short reflow causes the transformation of the core and the shell yielding a stable high melting point solder with adjustable melting points. The chosen ternary solder system enables the realization of interconnects with melting points in the range of 112 °C to 206 °C.

## Introduction

The ongoing trend in more intelligent, complex, and modern electronic systems requires advanced packaging technologies for a reliable integration of high performance inorganic semiconductor devices on desired substrates. While the traditional field of microelectronics deals with high functional density and fine-pitch integration, the field of macroelectronics focuses on the large-scale integration of dies on curved flexible, soft and even stretchable substrates^1,2^. The realization of reliable solder bump based interconnects between the electronic components and flexible and stretchable substrate materials is challenging. The reflow duration and temperature is generally restricted^3^.

Utilizing methods of integration range from robotic pick-and-place, parallel transfer^4,5^, and fluidic self-assembly^6–10^. The latest, solder directed fluid self-assembly enables a high level of parallel assembly, self-alignment and formation of electrical connections. The driving force behind this is the minimization of the surface energy of the molten solder-water interface^10^. The formation of solder bumps is therefore a particularly important factor in this assembly method. Previous works in this field employed methods of dip-coating^11^, electroplating, or a combination of both for the solder bump formation^12^. Since the solder volume affects the final assembly yield, dip-coating is challenging especially, when it goes to miniaturization, fine pitch and high-density assembly. Furthermore, it requires several steps of lithography and patterning. Another challenging point is the assembly medium, which is restricted to the boiling point of the medium and the melting point of the solder. Recent works demonstrated self-assembly in ethylene glycol on solder bumps with melting points higher than 100 °C^13^. We recently reported a core-shell transformation imprinted solder bump to enable mounting of chips at relatively low temperatures (<80 °C) while providing a route to stable high melting point interconnects (up to 206 °C) through transformation^14^. The method used a low melting point molten shell to capture and self-align chips during the mounting process at temperatures below 80 °C (in water). A short final reflow leads to diffuse the high melting core to the shell and to raise the melting point of the final alloy to desired levels. The bumps were applied as a novel receptor to capture agitated chips in heated (<80 °C) water bath. The bumps maintained the comparatively high assembly yields that have previously been reported in molten solder directed fluidic self-assembly^2,10,15–18^. Equally, high self-alignment properties were observed. As a new element, the final melting point was raised using the transformation imprinted properties. However, the recently published method used a dip coating step to apply the low melting point solder shell^14^. This step had some disadvantages. The solder volume could not be precisely adjusted, which leads to changes in the composition of the final alloy.

This publication introduces a one-step lithographic galvanic alternative to enable the production of multilayer solder bumps with tailored transformation imprinted melting points. The galvanic process provides a greater control over the composition of the individual solder bumps. Moreover, the route is no longer limited to two layers. Instead, multiple layers can be deposited. The conceptual approach will be demonstrated using a lead free ternary solder system containing bismuth, indium and tin with tailored melting points between 112 °C and 206 °C, which was not possible before. The utility of the bumps as receptor elements in fluidic self-assembly experiments will be demonstrated. During the self-assembly experiments the transformation imprinted properties serve different tasks. Specifically, the fluidic self-assembly process uses the molten low melting point shell (two outer metal layers) as a selective adhesive to capture chips inside a heated (80 °C) agitated water bath. During the capturing process, the molten metal wets the metal contact (binding side) on the agitated chips leading to captured, self-assembled, self-aligned, electrically connected, and well-ordered chip arrays. The solid cores (lowermost metal layer) serve as anchor points to the substrate during the self-assembly process. A final and short reflow process transforms the multilayer solder stack into a solid electrical connection with a higher and tailored melting point. An adjustable transition imprinted melting point between 112 °C to 206 °C is demonstrated.

## Materials and Methods

**Figure 1:**
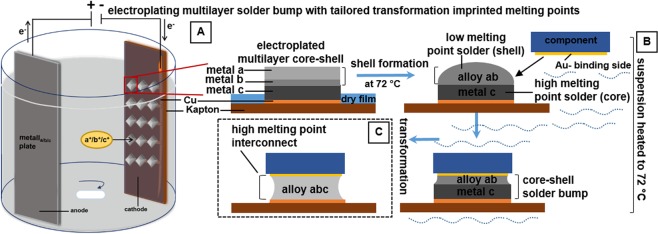
Electrodeposition of multilayer solder bumps with tailored transformation imprinted melting points used to assemble dies at 75 °C and create high melting point interconnects after transformation. (**A**) Electrodeposition bath containing array of copper-pads (receptor sites) on a flexible polyimide substrate, electrolyte, and the metal plate. The metal layers a, b, and c are deposited in separate electroplating baths. (**B**) The solder bumps are composed of a high melting point solder (core), metal layer c, and a low melting point solder (shell, alloy ab with MP 72 °C). The low melting point shell enables assembly at low temperatures. The fluidic self-assembly process is carried out in a assembly medium (water) at a temperature above the melting point of the shell. The solder wets the Au coated contact (binding side) on the die; reduction of the interfacial free energy of the molten solder ab drives the capturing and alignment process. (**C**) A short high temperature reflow step transforms the entire electrodeposited metal stack to a high melting point solid interconnect (alloy abc).

Figure 1 illustrates the formation of solder bumps through multilayer sequential electrodeposition of various base metals *a*, *b*, and *c*. The two outer metal layers, *a* and *b*, form the low melting point solder shell. This shell is essential since it enables assembly of dies at low temperatures. As an application, the shell is used as selective adhesive to capture and self-assemble agitated dies and surface mount devices in a modestly heated water environment. The lowermost metal layer ***c*** is the high melting point solder, the core, and is used in a final reflow step to increase the melting point of the interconnect. (A) The solder bump is fabricated through sequential layer-by-layer electroplating of three base metals. The desired solder composition and consequently the melting point of the solder is reached by adjusting the individual layer thickness. The shell is composed of two metal layers bismuth and indium (*a* and *b*), which form the low melting point shell at 72 °C (alloy *ab*). The electroplated core (*c*) is a higher melting point solder (>156 °C). The core remains solid during the assembly process and acts as an anchor point to the substrate. (B) During the self-assembly process the low melting point shell is molten and is used to capture and align the distributed parts in heated water (<100 °C) by wetting the metal contacts, the so-called binding sides, of the parts. The driving force behind this process is the reduction of the interfacial free energy of the molten solder layer. The wetting of the metal contact by molten solder and the formation of the solder bridge reduces the exposed solder-water interfacial area. Once the molten solder captures the chip, mechanical and electrical connection between the chip and substrate is provided. (C) A short reflow step causes the transformation of the entire metal stack to a high melting point interconnect by mixing of core metal with the solder shell.

The aim of this work and our previous work was to develop methods of self-assembly which enable chip capturing and alignment at fairly low temperatures (<100 °C). As an example, 75 °C is considered a low temperature from a soldering point of view. Typical soldering temperatures are >200 °C. Water is the solvent that is used in this study. Chips and wafers are commonly washed in DI water and are compatible with this solvent. Moreover, it can be used in combination with a fluxing agent to maintain an oxide free surface. We decided to use water since it is abundantly available and disposable.

Among different techniques used in bump fabrication such as solder ball bumping^19^, screen printing^3,8,20^, and dip-coating^13^, the illustrated electroplating was chosen and optimized, since it provides a clear path towards miniaturization, uniform bump size, and compositional multilayer control^21,22^. However, some challenges had to be overcome. Initial trials confirmed that co-deposition is challenging. The different metal contents of the plating solution varied during co-deposition trials and a constant alloy composition could not be obtained, this is a common problem^23,24^. The illustrated stack was fabricated by successive deposition of solder metal layers in separate baths. This was found to be the only working strategy. Even then, challenges had to be overcome. For example, electrodeposition of different metal layers with a large difference in standard electrode potential is another known challenge, which limits the range of materials that can be used. Additionally, the number of lead- and cadmium-free eutectics with a melting point below 80 °C is limited. Considering some of these constraints, the study identified a working solder system, which contained bismuth, indium and tin. As a low melting point shell we targeted the deposition of a Bi_33.7_In_66.3_-eutectic (MP.72 °C, Indalloy #162, Indium Corp, subscripts in wt%) to enable the assembly at temperatures well below a set 100 °C threshold. The core is used as a transformational element to shift the composition and to raise the melting point in the final structure. For example, diffusing bismuth-, indium- or tin-core metal into the Bi_33.7_In_66.3_-shell increases the melting point of the interconnects, as discussed later.

**Figure 2:**
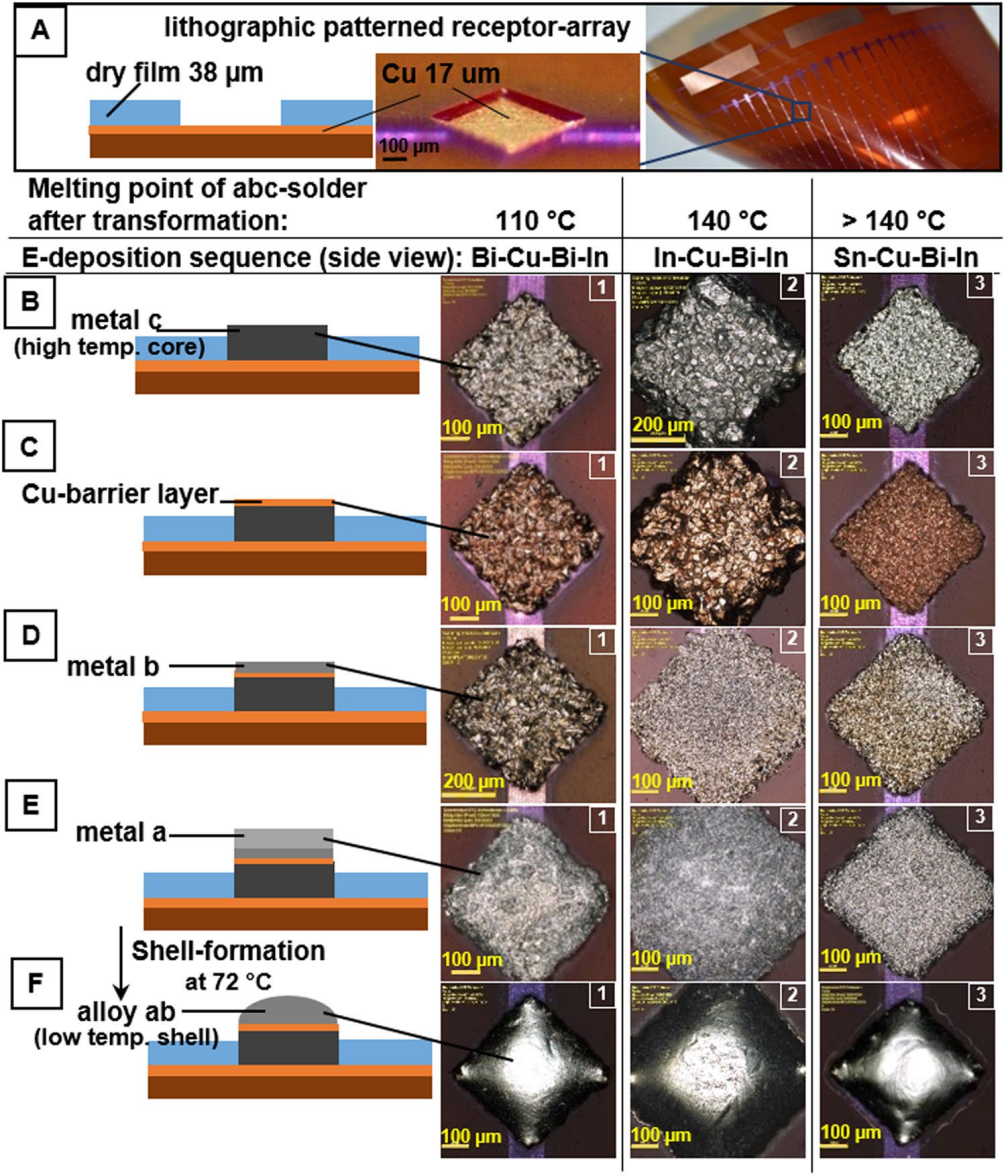
Schematic and photographs (Laser Scanning Microscope, LSM) illustrating the fabrication steps of electrodeposited multilayer solder bumps with different compositions of Bi, In, and Sn. (**A**) Lithographic patterning; array depicts 152 Cu-receptors (400 × 400 × 17 µm^3^) supported on a flexible Kapton (polyimide) substrate (9 × 5 cm^2^). The Cu-pads are connected via an interconnecting line. These lines are covered with a dry film solder mask (38 µm). (**B**) Core metal layer electroplating of bismuth (B_1_), indium (B_2_), and tin (B_3_); followed by (**C**) a thin (1 µm) copper intermediate layer to narrow the potential difference between the core and first shell layer. (**D**) Bismuth and (**E**) Indium electrodeposition, (**F**) forming the desired Bi_33.7_In_66.3_ (alloy ab) through reflow at 72 °C. This alloy is used to wets the metal binding site of the chip and drives the self-assembly process. The final composition of core-shell solder is controlled by adjusting the thickness of each metal layer by the deposition time.

Figure 2 describes the fabrication of electrodeposited multilayer solder bump with tailored transformation imprinted melting points in a one-step-lithographic fashion; (A) lithographic patterning of receptor array, electrodeposition of (B) core-metal, (C) Cu-intermediate, (D-E) shell-metal-layers, and (F) BiIn shell alloy formation.

(A) A copper-coated polyimide film (50 µm thick with 17 µm Cu, AKAFLEX^®^ KCl HT) is used as substrate. Each substrate was 9 × 5 cm^2^ in size and contained 152 assembly Cu-receptors. The 400 × 400 µm^2^ sized Cu-receptors are patterned by laminating a layer of dry film (38 µm, Vacrel® 8100, DuPont) on Cu-coated polyimide followed by steps of photolithography and Cu-etching. A second layer of dry film is applied to cover the interconnecting lines and create openings with a 400 × 400 µm^2^ sized copper pad. The openings are used to deposit the solder metal layers in subsequent steps. All electroplating steps are carried out in separate electroplating baths.
(B) (B) A short pickling of the Cu-pads is done in a 5% H_2_SO_4_ solution. Subsequently, the high melting point core is electroplated. Three base metals are tested, bismuth, indium, and tin.
  (*B*_*1*_ *- Bismuth Core - Left*) A solution of Bi(III)-methanesulfonic acid containing 210 g L^−1^ bismuth was applied for bismuth core electrodeposition. The deposition is kept at room temperature with a current density of 1 A dm^−2^.
  (*B*_*2*_ *- Indium Core - Center)* Indium is a base metal with a negative standard electrode potential, which leads to the simultaneous strong hydrogen deposition and challenges in electrodeposition. Furthermore, the common available alkaline In-electrolyte could not be applied, since the dry film is not resistant to alkaline solutions. Therefore an acidic In-electrolyte based on In(III)-methanesulfonic acid containing 120 g L^−1^ indium and sodium dodecyl sulfate (SDS) was developed. Sodium dodecyl sulfate is a surfactant that decreases the hydrogen bubble life time. This is more discussed in Supporting Information (S1). The indium core layer was deposited at 40 °C with a current density of 2 A dm^−2^ in an ultrasonic bath for a uniform deposition.
  (*B*_*3*_ *- Tin Core - Right*) The Sn-core layer was deposited using an acidic tin electrolyte NBT Semiplate Sn 100 (MicroChemicals, Ulm, Germany) containing 40 g L^−1^ tin, based on tin(II)-methanesulfonate and methanesulfonic acid. A current density of 1 A dm^−2^ was applied. Bi- and Sn-deposition is supported by stirring.
  To avoid contamination with sulfur (contained in the various electrolytes), cleaning with DI-water (immersion in a beaker for 1 minute after each step of electroplating) was used as a precaution. Sulfur residue leads to brittleness in connection to the substrate. The EDX-Investigation of shell solder showed in Supporting Information Fig. S2 confirms the lack of sulfur in the electrodeposited multilayer solder bump Bi_33.7_In_66.3_.
(C) A Cu-intermediate layer is deposited directly on top of deposited core metal layer. This layer was required to narrow the potential difference between core and first shell metal layers to reduce the negative effects of cementation. Tin and indium have a standard electrode potential of −0.137 V (Sn^2+^/Sn)^25^ and −0.342 V (In^3+^/In)^26^ and are less noble metals than bismuth (first shell metal layer) with +0.317 V (Bi^2+^/Bi)^27^. This leads to the cementation of bismuth on tin or indium during the galvanic deposition. The intermediate copper layer with a standard electrode potential of +0.340 V (Cu^2+^/Cu)^27^ eliminates this issue. However, the copper electrodeposition required extra care. The commonly used Cu-electrolyte is based on copper(II)-sulfate and sulfuric acid which did not work on tin and indium again due to cementation issues. A Cu-electrolyte (pH 8) based on organic phosphonates as complexing agent was developed to overcome this issue. The complexing agents shift the potential of copper to a more negative value so that no electroless copper deposition on tin or indium occurs. Additionally, the Cu-intermediate layer reduced the diffusion from the solid core to the liquid shell, which was found to be essential to accomplish stable and switchable melting points.

(D–E) The low melting point BiIn shell is electroplated in two sequential steps; bismuth first and indium next. We used the previously described indium (*B*_*2*_) and bismuth (*B*_*1*_) electrolyte and plating conditions. The eutectic composition is controlled by adjusting the thickness of each metal layer by deposition time. As mentioned, the successful sequential deposition required the Cu-intermediate layer. A direct galvanic deposition of Bi on In or Sn is not possible due to the potential mismatch.

(F) A short reflow step at 72 °C forms the uniform low melting point Bi_33.7_In_66.3_ solder bumps that enables the mounting of electronic components at low temperatures. These bumps are also used as receptors and selective adhesives to capture chips from an agitated heated water bath.

As previously mentioned, the low melting point BiIn shell is deposited in a two-step sequence. The thickness of the individual layers ranges between 10–30 µm, depending on the final composition of the transferred solder bump. The high diffusion rate of indium into bismuth leads to the formation of intermetallic layers in the following sequence: In, In_2_Bi, In_5_Bi_3_, InBi and Bi. This occurs even at room temperature. The diffusion continues until In_2_Bi and In_5_Bi_3_ phases are completely decomposed and a layer of InBi is formed^28^. The supplemental section provides SEM, EDX, and DSC investigations which confirms the target (Bi_33.7_In_66.3_) composition (S2). A XRD-diffractogram confirms that no Bi peek is present (complete diffusion has occurred) by an as-deposited sample (S3).

An A4 sized copper-coated polyimide (AKAFLEX® KCl HT, Germany) is the starting material to fabricate self-assembly substrate with Cu-receptors and interconnecting lines. First, the Cu layer (17 µm thick) was roughed mechanically with a bursting machine. Second, dry film (38 µm, Vacrel® 8100, DuPont) was laminated on the top using a tension controlled laminator at 120 °C with 250 kPa. Finally, the dry film was exposed with UV light through a transparency mask, and was developed in a solution of Na_2_CO_3_ at 28 °C for 3 min. A wet-chemical etching step in a solution of Na_2_S_2_O_8_ and H_3_PO_4_ at 50 °C for 4 min was applied to pattern the Cu structures. The dry-film was removed in surfacestrip™ 419 at 45 °C leaving Cu squares (receptors) and interconnecting lines on the substrates. The substrate was rinsed with DI water and dried with compressed air.

To demonstrate the self-assembly on electroplated multilayer solder bumps 500 μm in lateral size Si chips were fabricated. A 225 μm thick Si wafer (MicroChemicals, Ulm, Germany) was cleaned with a Piranha solution (1 H_2_SO_4_: 1 H_2_O_2_) at 120 °C for 15 min and rinsed with DI water. To pattern the Au-pads (400 × 400 µm^2^) on Si, photoresist (AZ 5214 E, MicroChemicals) was spun on at 2,000 rpm for 60 sec. After a soft baking for 1 min, the substrate was exposed through a transparency mask for 1.3 sec. Subsequently, the wafer was developed in 1:1 AZ-developer after a 2 min baking at 120 °C. An e-beam evaporator (CS400, Ardene) was used to coat the wafer with 10 nm Ti, 200 nm Cu, 200 nm Ni and 150 nm Au layers. Titanium is applied as adhesion metal layer. Since a thin Au-layer dissolves quickly in the molten high temperature solder, a Ni layer underneath supports the metallurgical bond. The lift-off process was succeeded in a solution of DMSO and cyclopentanone at 80 °C for 1 hour. Finally, the wafer was dried and diced using a wafer dicing saw.

For DSC investigations, a single receptor (400 µm × 400 µm) was cut at the receptor edges after steps of electroplating. The measurement was carried out under nitrogen and the sample was kept in a small aluminum crucible. A DSC 204 F1 Phoenix® ASC device was applied.

## Results and Disscution

**Figure 3:**
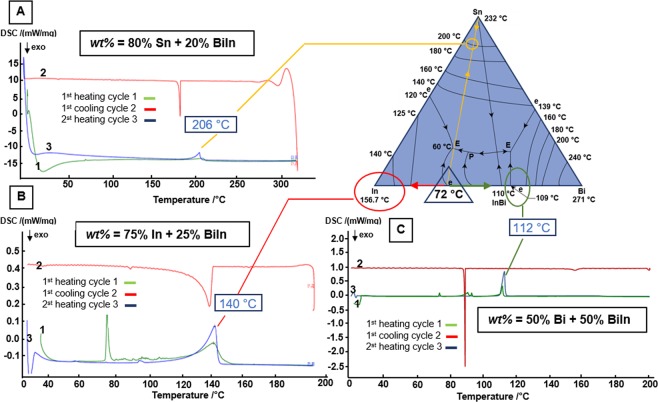
Tailored transformation imprinted melting points using electroplated multilayer solder bumps with corresponding ternary Bi-In-Sn phase diagram^29^ and differential scanning calorimetric (DSC) graphs, investigating three different core metals (**A**) tin, (**B**) indium and (**C**) bismuth. The 72 °C peak of the solder shell (Bi_33.7_In_66.3_) is visible in the first heating cycle (green curve) in all three DSC-graphs. The final melting point of the interconnect is visible in the second heating cycle (blue curve). (**A**) 80 wt% tin core transforms the 72 °C melting point to 206 °C after reflow. (**B**) 75 wt% indium core transforms the 72 °C melting point to 140 °C. (**C**) 50 wt% bismuth transforms the melting point to 112 °C.

Figure 3 depicts the possible routes to adjust the melting point of electrodeposited multilayer solder bump based on the ternary Bi-In-Sn phase diagram^29^ and Differential Scanning Calorimetry (DSC) measurement graphs. The blue triangle in the phase diagram highlights the low melting point eutectic BiIn composed of 33.7 wt% bismuth and 66.3 wt% indium with a melting point of 72 °C. This eutectic is used as the solder shell. The yellow, red, and green lines illustrate the trajectories of three different core metal compositions that we evaluated to enable the transformation of the final melting point.

(A) Applying 80 wt% tin as the high melting point solder underneath the BiIn shell (20 wt%, MP 72 °C) yields a solder joint with a melting point of 206 °C. The anticipated transformation can be followed in the DSC graph. The 72 °C peak of the Bi_33.7_In_66.3_ shell is visible in the first heating cycle (green curve 1). The peak disappears during the cooling cycle by recrystallization (red curve 2), which is a result of the complete diffusion of the shell in the core metal layer. A single peak by 206 °C during the second heating cycle (blue curve 3) confirms the formation of the higher melting point interconnect.
(B) Replacing tin with indium following the red trajectory in the phase diagram is a second option that we tested. Specifically, we used a 75 wt% electroplated indium as a core in combination with a 25 wt% electroplated BiIn shell. This core-shell combination transforms the 72 °C melting point to 140 °C after a short final reflow, as can be seen comparting the corresponding peaks in the DSC plots.
(C) Another adjustment is possible by electroplating 50 wt% bismuth core with 50 wt% BiIn-shell, following the green trajectory in the phase diagram. In this case, the final solder alloy has a melting point of 112 °C.

We would like to note that the final melting point of the solder bump is not restricted to the three demonstrated core-shell compositions. For an example, different weight fractions of Sn and BiIn, can be used to adjust the melting point to any value between 140 °C and 200 °C, regarding the phase diagram.

**Figure 4:**
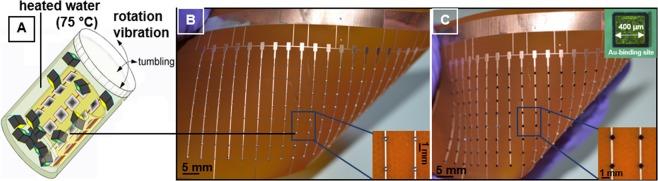
(**A**) Self-assembly schematics, (**B**) first results on electroplated multilayer solder bumps on large scale substrate after shell formation, (**C**) after self-assembly and melting point transformation (50 wt% B – 50 wt% Bi_33.7_In_66.3_). (**A**) Schematic of assembly barrel; rotation and vibration during the assembly process provides the distribution of the chips over the entire substrate; the assembly medium is heated to 75 °C, the molten low melting point shell captures and aligns chips. (**B**) Polyimide substrate with an array of 152 Cu-receptors coated with electroplated multilayer Bi-BiIn solder bump after the shell formation. (**C**) Photograph depicting correctly assembled Si-dies after self-assembly and transformation, no dies are missing.

Figure 4 presents the self-assembly results using the electrodeposited multilayer solder bumps. The self-assembly process was carried out in an assembly medium (water) at 75 °C. First, the molten solder shell wets and captures the metal contacts on the chips. Second, a final reflow step transforms the 72 °C melting point solder into a 112 °C – 206 °C melting point interconnect. The temperature value depends on the core material used. (A) The self-assembly was carried out in a barrel like assembly container at 75 °C. A video presenting a typical example of such a self-assembly experiment is provided in the Supporting Information. The flexible Kapton substrate is rolled and attached to the internal wall of the barrel. In order to obtain an oxide free surface during the assembly process, small amounts of HCl are added to the water to provide a 0.1 molar DI/HCl solution, which acts as a fluxing agent. The barrel contains approximately 1000 silicon dies with a dimension of 500 × 500 × 225 µm^3^. The self-assembly process requires agitation of the components and component delivery to the receptors. With a barrel rotation of 2 RPM and vibration of 2 cm amplitude at 5 Hz frequency the components are agitated in assembly medium. The agitation is required to distribute the components over the entire substrate. The assembly process is completed in 6 minutes; this duration includes 3 minutes of preheating. The actual experiments were carried out using the Bi_33.7_In_66.3_ shell with Sn-, Bi-, and In-core in separate tests. Qualitatively, we found no difference in terms of self-assembly yield. Moreover, we found no difference when compared to the dip-coated Bi_33.7_In_66.3_ solder (subscripts in wt%), which is commercially available (Indalloy #162, Indium Corp). Furthermore, 100% coverage and assembly yield is achieved. (B-C) provides a representative overview (B) of the receptor array using electroplated multilayer solder bumps after shell formation (C) and after self-assembly and transformation. In this experiment, we used the solder composition 50 wt% Bi – 50 wt% Bi_33.7_In_66.3_. A short (1 minute) reflow step at 120 °C was applied to transform the melting point of the bumps. The final bumps had a melting point of 112 °C, which was discussed by means of DSC-graph earlier.

Generally, all three compositions of BiIn-shell with Bi, In, and Sn core worked equally well in terms of electrodeposition, shell formation, capturing, self-assembly and self-alignment, and transformation step.

**Figure 5:**
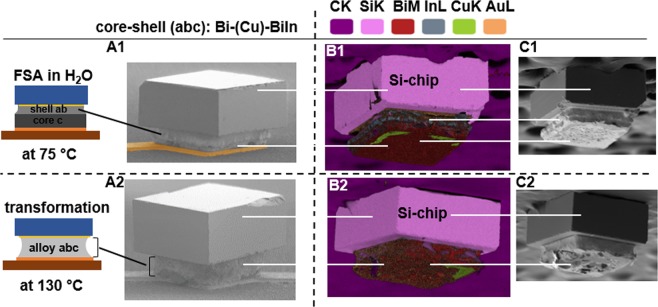
Schematics, scanning electron micrographs (SEM) of a captured and self-aligned chip (**A**) and energy-dispersive X-ray spectroscopy (EDX) of a Si-component detached from the Cu-receptor pad before and after transformation (**B**,**C**). (A1) and (A2) SEM of a single captured, self-assembled, and self-aligned chip before and after transformation of the melting point; the 400 µm × 400 µm pad on the dies aligns to the 400 µm × 400 µm copper receptor on the substrate. (B1) EDX investigation of the detached Si-chip; core and shell layers are separated via the Cu-intermediate layer. (B2) A uniform distribution of the three metal atoms Bi, In, and Sn, and the formation of the final high melting point solder join is to observe. The Cu-intermediate layer is no longer to observe. (C1) and (C2) SEM images of the detached chips.

Figure 5 provides the (A) scanning electron micrographs (SEM) of a self-assembled and self-aligned chip on a Bi-BiIn electroplated solder bump and (B) energy-dispersive X-ray spectroscopy (EDX) of such a solder bump from a detached chip before and after the transformation. EDX helps to observe the distribution of atoms in different electrodeposited metal layers of the electroplated solder metal stack. (A1-A2) Scanning electron micrograph of a single captured and assembled die before and after the transformation. While the interface between core and shell solder layers is visible in the first image (A1) a uniform bump is formed after the final reflow and transformation (A2). In this experiment, we used a reflow step of 1 minute at 120 °C to transform the melting point of the bumps. The final solder bumps had a melting point of 112 °C and a mass ratio of 50% Bi – 50% Bi_33.7_In_66.3_. (B) In order to observe the distribution of different atoms of the electrodeposited multilayer core-shell after self-assembly and after the transformation a captured and aligned component was detached from the Cu-receptor after every step. EDX investigation was carried out on the solder bump for the both samples. (B1) Diffusion of two outer metal layers (a and b) Bi and In forms the low melting point shell (red and gray), which is clearly separated from the core in Fig. C1. There are some residue of Cu-pad (green) on the shell to observe. (B2) A uniform distribution of three metals Bi, In, and Sn can be observed after the transformation step. The intermediate layer, which acts as a barrier layer between core and shell can no longer be observed (C2). The homogenous distribution of the different metal atoms confirms the formation of the final high temperature solder joint, which was discussed in Fig. 3C using the DSC measurement graph.

We used a single lithography with a thick film (38 µm) solder mask for all galvanic deposition steps to protect the interconnecting lines and define the electrodeposition window on Cu-pads. Since the last electrodeposited layers are grown over 38 µm solder mask sidewalls, it is challenging to observe the core metal under the shell solder in EDX investigations even with a tilted angel. After the temperature transformation, the metal layers form a unique solder bump.

## Conclusion

The electroplated multilayer solder bumps with tailored transformation imprinted melting points provide a new method to engineer the melting point of the interconnects. The solder bumps enable fluidic self-assembly and self-alignment at 75 °C and enable the tailored transformation imprinted solder based interconnects with melting points between 112 °C and 206 °C in the final composition. The sequential electroplating approach enables a greater control over the composition when compared to previous work. Moreover, the volume is reduced by about a factor of 0.6. This has a potential for applications with fine pitch and high density solder bumps. Future research should look at variation of solder volume on self-assembly and alignment process. Additionally, mechanical and electrical properties of different compositions should be studied. In addition, the presented multilayer solder bump electroplating shows potentials in self-assembly and self-alignment of chips with more than one contact pad, since solders with different melting points can be deposited sequentially.

## Supplementary information

Dataset 1

Self-Assembly video
