# Supplementary material for: Fluidic Self-Assembly on Electroplated Multilayer Solder Bumps with Tailored Transformation Imprinted Melting Points
Source: Sci Rep. 2019 Aug 5;9:11325. doi: 10.1038/s41598-019-47690-8 (PMC6683123; doi:10.1038/s41598-019-47690-8)
Supplement: Supplementary file 1 — Dataset 1 [file 41598_2019_47690_MOESM1_ESM.pdf]

SUPPORTING INFORMATION

Fluidic Self-Assembly on Electroplated Multilayer Solder Bumps with Tailored Transformation Imprinted Melting Points

*Mahsa Kaltwasser[1], Udo Schmidt[2], Lars Lösing[2], Shantonu Biswas[†1], Thomas Stauden[1], Andreas Bund[2], Heiko O. Jacobs[1]**

[1] Fachgebiet Nanotechnologie, Technische Universität Ilmenau, Gustav-Kirchhoff-Strasse 1, Ilmenau D-98693, Germany, E-Mail*: heiko.jacobs@tu-ilmenau.de, +49 3677/69 3723

[2] Fachgebiet Elektrochemie und Galvanotechnik, Technische Universität Ilmenau, Gustav-Kirchhoff-Strasse 6, Ilmenau D-98693

[†]California NanoSystems Institute, University of California, Santa Barbara, CA 93106, USA

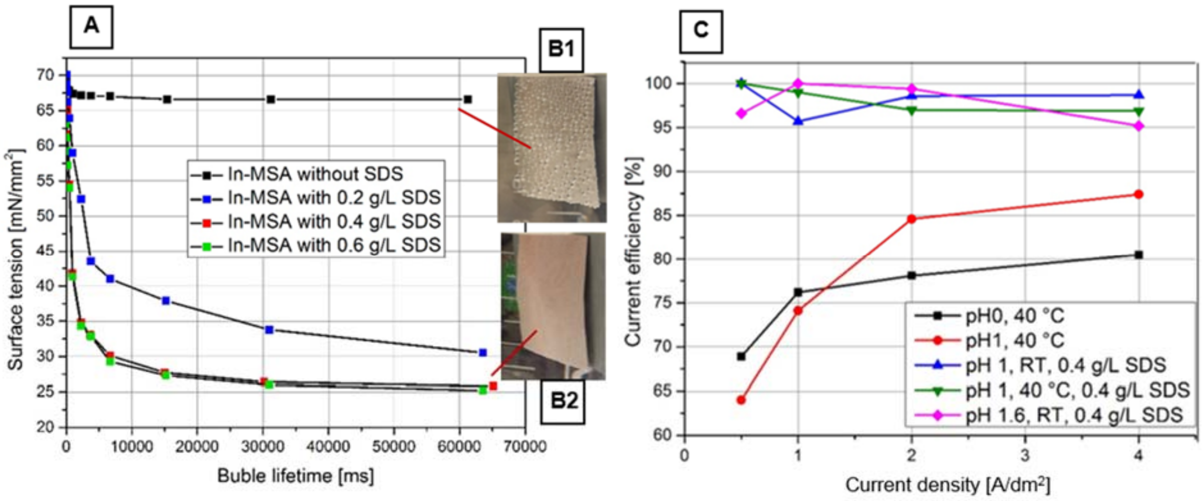

Figure S1 Optimization of developed indium electrolyte based on In(III)-methanesulfonic acid containing 120 g L^{-1} indium. (A) Adding 0.4 – 0.6 g L^{-1} Sodium dodecyl sulfate (SDS) to the In(III)-methanesulfonic acid decreases the life time of hydrogen bubbles on the surface. (B1) Without SDS and (B2) with SDS electrolyte shows a significant difference in the life time of formed hydrogen bubbles on the In-surface during the electrodeposition. The hydrogen bubbles prevent the deposition of In-metal atoms locally and leads to a rough and inhomogeneous deposited metal layer. (C) Adjusting the pH-value to 1 and deposition temperature to 40 °C, a current efficiency of 100 % was achieved with a current density of 2 A dm^{-2}.

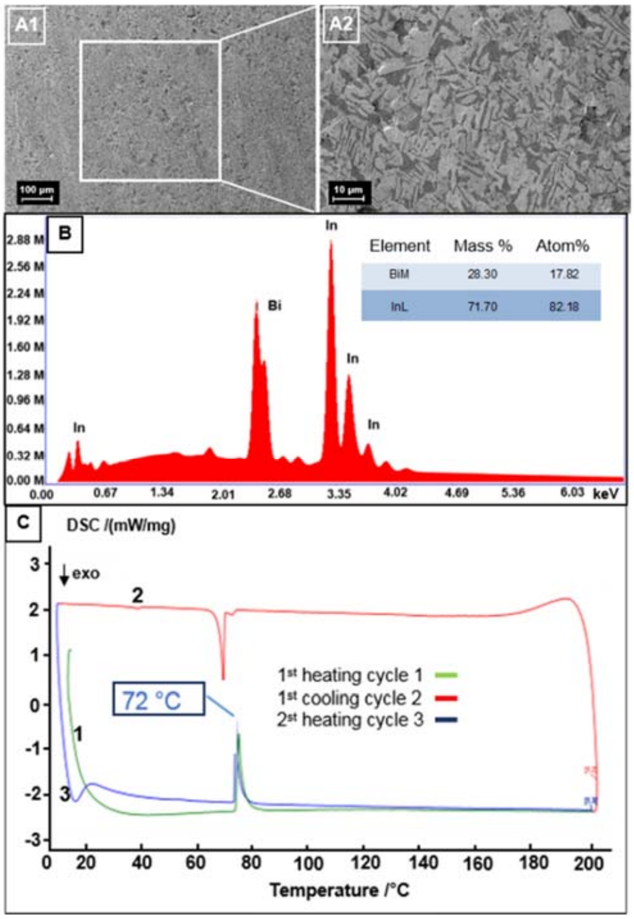

Figure S2 SEM, EDX, and DSC analyses of galvanic deposited BiIn-shell solder alloy. (A1) SEM image of the surface of electrodeposited $Bi_{33.7}In_{66.3}$ shell solder after the reflow at 72 °C. A 17 µm Cu-pad was used as a substrate to deposit the desired bismuth and indium amount in separate bath, successively. After deposition of 7 µm Bi and 23 µm In, a short reflow at 72 °C forms the eutectic $Bi_{33.7}In_{66.3}$ (Indalloy #162, Indium Corp, subscripts in wt%). (A2) Full-scan-X-ray mapping of an area of 50 µm x 50 µm (512 pixel x 513 pixel) of the surface of reflowed sample. Two phases are to observe; first the bismuth rich intermetallic $BiIn_2$ phase with 61.1% Bi and 38.9% In (wt%). Second, an indium rich phase with 96.9 wt% In. (B) EDX-analysis shows an alloy composition of 28.30 wt% Bi and 71.70 wt% In. (C) The single peak at 72 °C during the first and second heating cycle (green and blue curve) confirms the exact amount of electrodeposited metal layers Bi and In which form the desired eutectic $Bi_{33.7}In_{66.3}$. The formation of the eutectic BiIn starts probably during the In-deposition at 40 °C by diffusion of indium atoms to the bismuth layer.

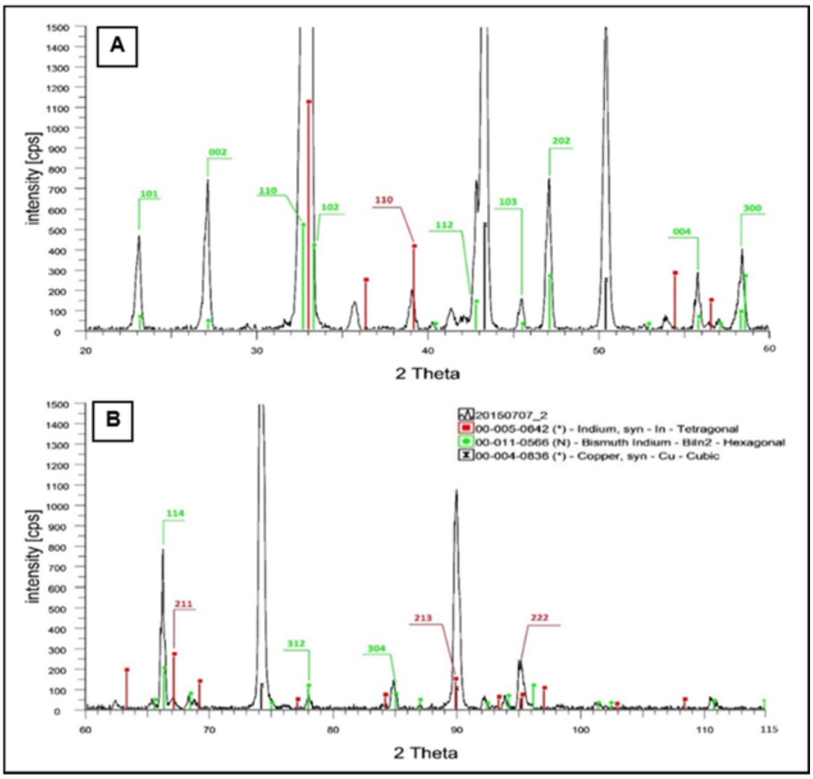

Figure S3 XRD investigation, ((A) 2-Theta scale 20 – 60 and (B) 60 – 115) of an as-deposited BiIn sample to confirm the diffusion rate of indium atoms into the Bi-layer. A 17 µm Cu-pad was used to deposit a Bi-layer with a thickness of 0.6 µm and an In-layer with a thickness of 1.9 µm on top. The In-deposition kept at 60 °C. The diffractogram shows three phases: In, Cu and $BiIn_2$. There is no peak of Bi to observe which confirms the high diffusion rate of In and the formation of the $Bi_{33.7}In_{66.3}$ solder directly after the deposition.

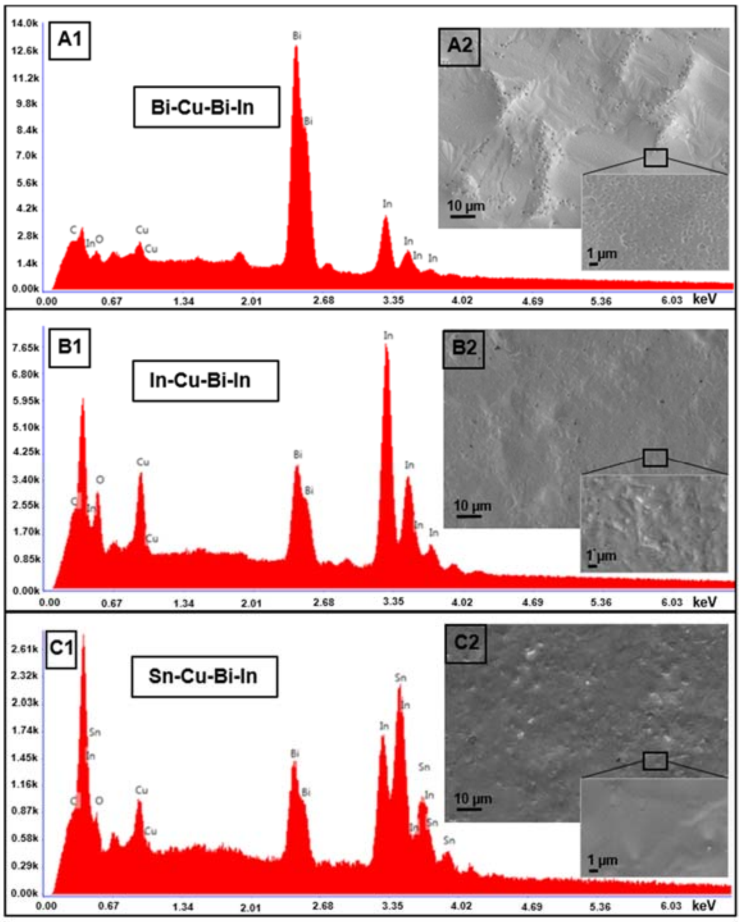

Figure S4 EDX analysis and SEM investigation of the surface of alloys abc after the melting point transformation. (A1) X-ray-scan of an area of 60 µm x 60 µm of electroplated multilayer Bi-Cu-Bi-In solder after the melting point transformation and (A2) SEM microstructure images of the scanned area. (B1) X-ray-scan of the electrodeposited multilayer In-Cu-Bi-In solder and (B2) SEM microstructure images of the scanned area. There is a significant difference in the Bi and In concentration between electrodeposited multilayer solder bumps with Bi-Cu-Bi-In and In-Cu-Bi-In composition to observe. (C1) X-ray-scan analysis of electroplated multilayer Sn-Cu-Bi-In solder and (C2) SEM microstructure images of the scanned area. It is to consider that Sn peak with corresponding binding energy of 3.443 keV and In peak with corresponding binding energy of 3.286 keV are partly overlapped. The Cu-peak is generated from Cu-intermediate layer but also from Cu-adhesive strip that was applied to fix the sample during the EDX-analysis.

Figure S5 cross sectional microscope images of electroplated multilayer solder (A1) Bi-Cu-BiIn, (C1) Sn-Cu-BiIn after the low melting point shell ($Bi_{33.7}In_{66.3}$, MP. 72 °C) formation. The intermediate Cu-layer is visible between low melting point solder (shell) and high melting point core. (B1) Since indium is a soft metal, it is not possible to prepare a polished cut before temperature transformation. Cross sectional microscope and SEM images of electroplated multilayer solder (A2-A3) Bi-Cu-BiIn, (B2-B3) In-Cu-BiIn and (C2-C3) Sn-Cu-BiIn after a short final reflow and melting point transformation. The Cu-intermediate layer (1 µm) is dissolved in entire solder bump after the transformation. The SEM images A3 and B3 confirm the crystal mixture morphology of bismuth and indium compositions.

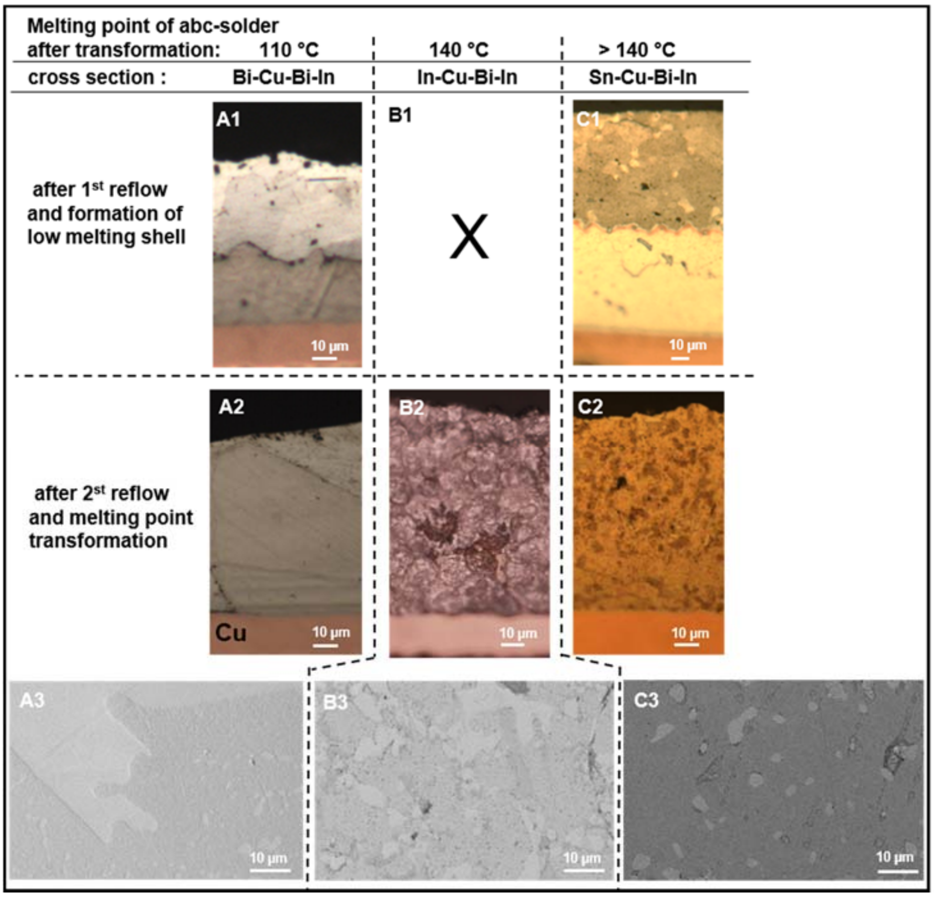

Figure S6 Delta Average Step Height (ASH) of five different located electroplated multilayer solder bumps Bi-(Cu)-Bi-In after the shell formation measured by Dektak profilometer. The five measured solder bumps are distributed over the entire substrate surface with a vertical distance of about 14 mm to each other. The measured ASH values range between 151 µm and 154 µm, which confirms a homogenous electrodeposition of the individual layers over the entire substrate (9 x 5 cm^2). It is to consider that the ASH values include the 17 µm thick Cu-receptor pad.

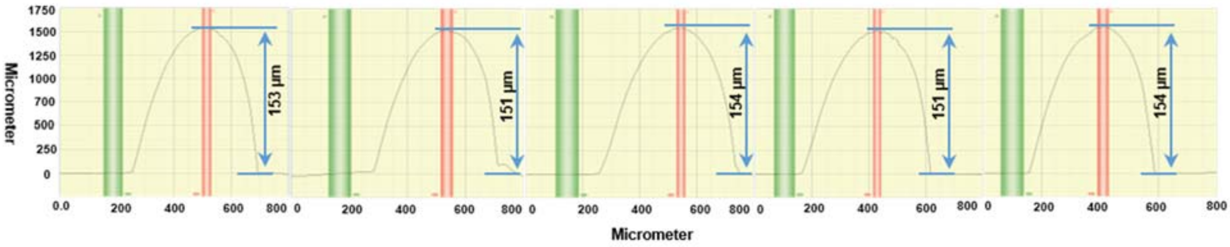

Movie: Fluidic Self-Assembly
